# Characterizing the neurocognitive profiles of children with moyamoya disease using the Das Naglieri cognitive assessment system

**DOI:** 10.1038/s41598-022-07699-y

**Published:** 2022-03-07

**Authors:** Yusuke Kusano, Takeshi Funaki, Keita Ueda, Noyuri Nishida, Kanade Tanaka, Susumu Miyamoto, Shuichi Matsuda

**Affiliations:** 1 Advanced Occupational Therapy, Department of Human Health Sciences, Kyoto University Graduate School of Medicine, 53 Kawahara-cho Syogoin Sakyo-ku, Kyoto, Japan; 2 Rehabilitation Unit, Kyoto University Hospital, 54 Kawahara-cho Syogoin Sakyo-ku, Kyoto, Japan; 3 Department of Neurosurgery, Kyoto University Graduate School of Medicine, Kyoto, Japan; 4 Department of Psychiatry, Kyoto University Graduate School of Medicine, Kyoto, Japan; 5 Department of Orthopedic Surgery, Kyoto University Graduate School of Medicine, Kyoto, Japan; 6 Faculty of Health Sciences, Department of Medical Welfare, Kyoto Koka Women’s University, Kyoto, Japan

**Keywords:** Neuroscience, Psychology, Medical research

## Abstract

Although cognitive impairment is well-documented in children with moyamoya disease (MMD), selective decline in specific neurocognitive domains remains controversial. The purpose of this study was to characterize the neurocognitive profile of children with MMD using the Das Naglieri Cognitive Assessment System (CAS) and the Wechsler Intelligence Scale for Children, Fourth Edition (WISC-IV). We analyzed the neurocognitive data of 30 children (median age, 7 years) with MMD who were assessed with the CAS and the WISC-IV before surgery. We focused on the comparison of standard scores and intraindividual differences across domains. The CAS scores significantly varied across four measures (standard scores, *p* < 0.001; intraindividual differences, *p* < 0.001). Post-hoc analyses revealed that the standard scores and intraindividual differences for successive processing were significantly lower than those for planning and attention. The WISC-IV scores did not significantly vary among the four measures, although the working memory index was the lowest among the four measures. The within-individual weakness in successive processing, a form of working memory function, may be a distinct characteristic of children with MMD. The CAS may be more sensitive than the WISC-IV for detecting this selective neurocognitive weakness in children with MMD.

## Introduction

Moyamoya disease (MMD) is a rare steno-occlusive cerebrovascular disease that involves the terminal portion of the bilateral internal carotid arteries. The age of onset of MMD has a bimodal peak: a major peak in the first decade of life and a moderate peak in the late 20s to 30s^1^.

Children with MMD commonly manifest ischemic symptoms, such as transient ischemic attacks (TIA) and stroke. However, children with MMD can also suffer from cognitive impairment. According to a recent systematic review, the prevalence of cognitive impairment is 30% (range 13% to 67%), and median intelligence quotient (IQ) is 98 (range 71 to 107)^2^. Long-term follow up studies have revealed that 10% to 20% of children with MMD have difficulty with social adaptation after adolescence because of cognitive impairment^3–7^. Such neurocognitive impairment is attributable to hypoperfusion in the vascular territories, which include the anterior and middle cerebral artery territories^1,8^ and the frontal lobe in particular^9–15^. Several multicentered registration studies, such as the Cognitive Dysfunction Survey of the Japanese Moyamoya Disease study^16^, are ongoing and aim to reveal neurocognitive and neuroradiological characteristics of moyamoya disease.

Although the mechanisms underlying neurocognitive impairment in moyamoya disease suggest selective impairment of specific neurocognitive domains that correspond to the vascular territories, the evidence remains controversial^2,17^. One possible reason is that the neuropsychological tests that are applicable to children are limited (e.g., the Wechsler Intelligence Scale for Children [WISC], the Das Naglieri Cognitive Assessment System [CAS], and the Kaufman Assessment Battery for Children). To date, there have been no studies conducted in children with MMD that have applied various neuropsychological tests on a large, representative, and standardized sample. Another possible reason for the controversy is that the interpretations of most available neuropsychological tests are based on comparisons with the normal population, which may underestimate the variability of neurocognitive domains within an individual.

The CAS is a standardized neuropsychological test developed by Das and Naglieri that focuses on the assessment of cognitive function, rather than intelligence, in children aged 5 to 17 years^18^. The official Japanese version of the CAS was developed in 2007^19^. The CAS comprises four measures: planning, attention, simultaneous processing, and successive processing. It is unique in that it assesses “intraindividual differences,” which are statistical fluctuations (strengths and weaknesses) across measures within an individual^20^. For this reason, we routinely use CAS as a preoperative neuropsychological test, in addition to the WISC.

The WISC is the most commonly used neuropsychological test worldwide that measures the intellectual ability of children aged 5 to 16 years^21^. The official Japanese version of the WISC fourth edition (WISC-IV) was developed in 2010^22^. The WISC-IV comprises four indices: verbal comprehension, perceptual reasoning, processing speed, and working memory. The WISC-IV implements a clinical method known as “the discrepancy analysis,” which aims to detect significant discrepancies among indexes in each individual. Kazumata et al. reported that discrepancies were most frequently observed between the verbal comprehension index and working memory index of the WISC-IV^23^. However, the discrepancy analysis of the WISC-IV is not necessarily suitable as a research method for detecting cognitive variability within a specific population. This is because, although discrepancy analysis of the WISC-IV only detects differences between each index, the CAS allows analyses of whether an index deviates from the average value of the entire individual. Therefore, the method whereby intraindividual differences are detected is limited in the WISC-IV.

To date, there have been no studies that have examined the neurocognitive function of children with MMD using both the CAS and the WISC. Determining whether specific neurocognitive weaknesses exist in children with MMD would be valuable for clinicians and rehabilitation professionals who support children with MMD.

The purpose of this study was to characterize the neurocognitive profile of children with MMD before surgery using the CAS and WISC-IV. We compared the scores of these neuropsychological tests and analyzed the intraindividual differences derived from these tests.

## Methods

This cross-sectional study was approved by the ethics committee of Kyoto University Graduate School, Faculty of Medicine (R1417). Study procedures were carried out in accordance with the Declaration of Helsinki. For prospective data, informed assent was obtained from all individual participants and written informed consent was obtained from their parents. For retrospective data, all subjects gave opt-out consent.

### Patients

The inclusion criteria were children aged 5 to 16 years who had been newly diagnosed with MMD and had undergone neuropsychological testing before surgery. Patients were diagnosed according to the criteria proposed by the Research Committee on Moyamoya Disease in Japan^1^. All moyamoya children aged 5 to 16 years and their parents were asked at their first visit whether they were willing to undergo neuropsychological tests.

A total of 40 children aged 5 to 16 years were admitted to Kyoto University Hospital between June 2016 and June 2020 and diagnosed with MMD. Of the 40 patients, 10 were excluded from the analyses for the following reasons: the patients and family declined the neuropsychological tests (2 patients); preoperative neuropsychological tests were not completed because of unstable symptoms (2 patients), limited examination schedule (1 patient); deafness (1 patient); lack of Japanese ability (1 patient); judgment by the psychiatrist that the neuropsychological test results were biased because of the patient’s developmental background (2 patients); and special education for gifted children (1 patient). This resulted in a total of 30 children for the analysis.

Clinical data were collected, which included sex, initial manifestations (e.g. TIA, stroke, intracranial hemorrhage, and headache), age at symptom onset and first admission, time interval between onset and first admission, Suzuki’s angiographical stage^24^, hemodynamic state assessed using single photon emission computed tomography (SPECT)^25^, laterality of disease involvement, presence or absence of posterior cerebral artery (PCA) involvement, and asymptomatic infarction on magnetic resonance imaging (MRI). PCA involvement was defined as stenosis greater than 50% in the P1 to P3 segment of either PCA, with decreased delineation of cortical arteries in either hemisphere^6^. The hemodynamic state for each hemisphere was graded according to SPECT findings into one of the following three stages: stage 0, normal baseline cerebral blood flow (CBF) with normal acetazolamide-challenged CBF; stage 1, normal baseline CBF with reduced acetazolamide-challenged CBF; and stage 2, reduced baseline CBF with reduced acetazolamide-challenged CBF^25^. Acetazolamide-challenged SPECT was not indicated for patients with severely reduced CBF in at least one hemisphere because of the risk of ischemic complication. Because this resulted in unclear differentiation between stages 0 and 1 in several hemispheres, we recorded hemodynamic state as either stage 2 or non-stage 2.

### Neurocognitive evaluation

The CAS and the WISC-IV were performed as preoperative neurocognitive assessments. All but one participant underwent both tests during the first admission. Occupational therapists, who are proficient in neurocognitive testing, conducted all tests in a consistent manner. Table 1 shows the comparison of characteristics between the CAS and WISC-IV.

**Table 1:** Characteristics of the CAS and WISC-IV.

	CAS	WISC-IV
Measurement	Cognitive processing	Intellectual ability
Age range	5:0–17:11	5:0–16:11
Publication date in Japan	2007	2010
Scores	Full scale Planning Attention Simultaneous processing Successive processing	Full scale IQ Verbal comprehension Perceptual reasoning Working memory Processing speed
Theory	PASS theory	CHC theory
Mean of standard scores	100	100
Standard deviation	15	15
Number of subtests	12	10 (primary) 5 (supplemental)
Number of standardization sample in Japan	1201	1293
Assessment of specific cognitive processing	Yes	No
Assessment of crystallized knowledge	No	Yes

*CAS* Das Naglieri cognitive assessment system, *CHC* Cattell-Horn-Carroll, *IQ* intelligence quotient, *PASS* planning*-*attention*-*simultaneous*-*successive, *WISC-IV* Wechsler intelligence scale for children, fourth edition.

The CAS provides standard scores for each of the four scales of planning, attention, simultaneous processing, and successive processing (PASS) as well as a full scale score^18^. The standard scores are defined by a mean of 100 with a standard deviation of 15. Each PASS scale comprises three tasks, with the whole battery comprising 12 tasks. The intraindividual difference is calculated by subtracting the average of the four PASS standard scores from each PASS standard score^20^. The intraindividual difference is negative if an individual scores relatively low in one PASS scale compared with the other scales. To interpret the CAS, a significant level of intraindividual difference was defined for each measure based on a standardized sample^19^. According to the manual of the CAS and WISC-IV, we defined a significant intraindividual difference as − 11 points or lower.

The WISC-IV provides standard scores for the following measures: full scale IQ, verbal comprehension index (VCI), perceptual reasoning index (PRI), working memory index (WMI), and processing speed index (PSI)^21^. These standard scores are defined by a mean of 100 with a standard deviation of 15 and are based on a large and representative standardized sample. Unlike the CAS, the calculation of the intraindividual difference is not specified in the WISC-IV. However, we used the same method as the CAS for calculating intraindividual differences based on the WISC-IV for the purpose of performing analyses within each participant.

### Statistical analysis

Wilcoxon rank-sum test was used for comparison between groups with and without asymptomatic infarction for mean full scale CAS and WISC-IV scores.

Descriptive statistics of the CAS and the WISC-IV are presented as mean and standard deviation for both the standard scores and intraindividual differences. Normality of the data was evaluated using the Shapiro–Wilk test. To compare the standard scores across the four measures of the CAS and the WISC-IV within each individual, we used a one-factor repeated measures analysis of variance (ANOVA). To compare intraindividual differences across the four measures, we used a one-way ANOVA because it was calculated based on the average of the four measures and thus, a repeated analysis was not necessary. Post-hoc tests were performed using Tukey’s honestly significant difference (HSD) test.

All statistical analyses were performed using JMP 14 (SAS Institute Inc., Cary, NC, USA). All tests were two-sided, and *p* < 0.05 was considered to indicate statistical significance.

## Results

### Clinical characteristics

Table 2 shows the clinical characteristics of the 30 included and 10 excluded patients. For the included patients, the median age of first admission was 7 years (interquartile range, 5.75 to 10 years) and the median time interval between symptom onset and admission was 6 months (interquartile range, 1 to 19.5 months). Females accounted for 70% of the included patients. The most common initial manifestation was TIA, which accounted for 73.3% of the included patients. One patient with stroke exhibited cortical laminar necrosis in the left primary motor cortex, which was confirmed by MRI, and mild paralysis of the right lower extremity, from which the patient made a full recovery. Three patients exhibited MRI findings of past intraventricular hemorrhage with no apparent intraparenchymal damage. Although hemorrhagic stroke caused consciousness disturbance during the acute phase, all three patients recovered without any neurological deficit before undergoing neuropsychological tests. Eleven patients (36.7%) had stage 2 hemodynamic failure in the right hemisphere, and 10 patients (33.3%) had stage 2 hemodynamic failure in the left hemisphere. Sixteen patients (53.3%) had stage 2 hemodynamic failure in at least one hemisphere. Asymptomatic infarction was observed in 11 patients (36.7%), which included the three patients mentioned above who exhibited hemorrhagic presentation.

**Table 2:** Participants’ clinical characteristics.

Variable	Inclusion (n = 30)	Exclusion (n = 10)
Median age at onset (IQR)	6 (5–9.25)	9.5 (5–11.25)
Median age at admission (IQR)	7 (5.75–10)	6 (0.46–13)
Interval btw onset and admission (mos)	6 (1–19.5)	14 (2.5–34)
Female (%)	21 (70)	5 (50)
**Initial manifestation (%)**		
TIA	22 (73.3)	8 (80)
Headache	4 (13.3)	1 (10)
Intracranial hemorrhage	3 (10)	0 (0)
Stroke	1 (3.3)	1 (10)
**Median Suzuki stage (IQR)**		
Right	3 (2–3)	3 (1–3)*
Left	2.5 (1.75–3.25)	2 (0–2.5)*
**SPECT stage in right hemisphere (%)**		
0 or 1	19 (63.3)	7 (70)
2	11 (36.7)	3 (30)
**SPECT stage in left hemisphere (%)**		
0 or 1	20 (66.7)	6 (60)
2	10 (33.3)	4 (40)
**Disease involvement (%)**		
Bilateral ICA	22 (73.3)	5 (50)
Unilateral ICA (right)	5 (16.7)	3 (30)
Unilateral ICA (left)	3 (10)	2 (20)
PCA involvement (%)	2 (6.7)	0 (0)
**Infarction (%)**		
None	19 (63.3)	4 (40)
Asymptomatic	11 (36.7)	6 (60)

*ICA* internal carotid artery, *IQR* interquartile range, *MMD* moyamoya disease, *PCA* posterior cerebral artery, *SPECT* single photon emission computed tomography, *TIA* transient ischemic attack.

*The data were missing in one patient because the patient did not undergo cerebral angiography.

### Neurocognitive findings

Mean full scale scores of CAS and WISC-IV were not statistically different between patients with and without asymptomatic infarction (CAS, *p* = 0.904; WISC-IV, *p* = 0.749).

Table 3 shows the descriptive statistics for each measure of the CAS and WISC-IV. All standard scores and intraindividual differences were normally distributed according to the Shapiro–Wilk test. The mean full scale scores of both the CAS and WISC-IV were within the normal range (102.2 ± 13.7 and 107.1 ± 14.4, respectively). The mean standard scores of the four separate measures in the CAS and the WISC-IV were also within the normal range, based on the standardized sample, except for planning (112.0 ± 13.3). In the CAS, scores for successive processing were the lowest among the four measures in terms of both standard scores and intraindividual differences (98.5 ± 14.1 and − 6.9 ± 8.7, respectively). In the WISC-IV, the WMI was the lowest among the four measures in terms of both standard scores and intraindividual differences (99.6 ± 13.7 and − 1.7 ± 8.7, respectively).

**Table 3:** Descriptive statistics and statistical analysis of the neurocognitive tests (n = 30).

Tests	Mean	SD	*F*	*P* value
CAS standard score*			9.206	< 0.001
Planning	112.0	13.3		
Attention	108.0	15.2		
Simultaneous	103.0	13.6		
Successive	98.5	14.1		
**CAS intraindividual difference**^†^			12.275	< 0.001
Planning	6.6	6.3		
Attention	2.7	11.2		
Simultaneous	− 2.3	9.9		
Successive	− 6.9	8.7		
WISC-IV standard score*			0.621	0.603
Verbal comprehension	100.6	15.1		
Perceptual reasoning	101.6	14.0		
Processing speed	103.3	13.2		
Working memory	99.6	13.7		
**WISC-IV intraindividual difference**^†^			0.828	0.481
Verbal comprehension	− 0.7	9.6		
Perceptual reasoning	0.4	9.3		
Processing speed	2.1	11.0		
Working memory	− 1.7	8.7		

*CAS* Das Naglieri cognitive assessment system, *WISC-IV* Wechsler intelligence scale for children, fourth edition, *ANOVA* analysis of variance.

*One-factor repeated measures ANOVA was applied.

^†^One-way ANOVA was applied.

**Figure 1:**
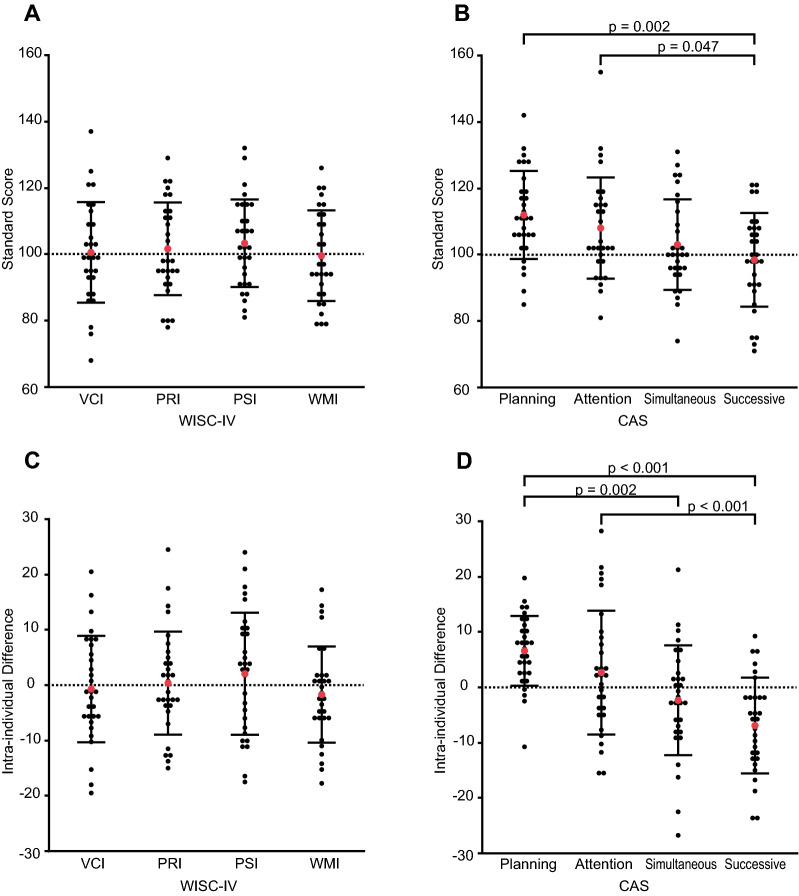
Graphs showing the neurocognitive data of each participant. **(A)** Standard score of the WISC-IV. **(B)** Standard score of the CAS. **(C)** Intraindividual difference in the WISC-IV. **(D)** Intraindividual difference in the CAS. The red dot indicates the mean value, and the error bar indicates the standard deviation. *CAS* cognitive assessment system, *PRI* perceptual reasoning index, *PSI* processing speed index, *VCI* verbal comprehension index, *WISC* Wechsler intelligence scale for children, *WMI* working memory index.

There was a statistically significant variance across all four measures of the CAS for standard scores and intraindividual differences, as shown in Table 3 and Fig. 1. The one-factor repeated measures ANOVA revealed a significant difference in the standard scores of the CAS (*F* (3,87) = 9.206, *p* < 0.001), but not in that of the WISC-IV (*F* (3,87) = 0.621, *p* = 0.603). The one-way ANOVA also revealed a significant difference in the intraindividual differences of the CAS (*F* (3,116) = 12.275, *p* < 0.001), but not in that of the WISC-IV (*F* (3,116) = 0.828, *p* = 0.481).

Table 4 shows the post-hoc multiple comparison of the CAS scores. Tukey’s HSD test applied to the CAS scores revealed that both the standard score and intraindividual difference of successive processing were significantly lower than those of planning (standard score, *p* = 0.002; intraindividual difference, *p* < 0.001) and attention (standard score, *p* = 0.047; intraindividual difference, *p* < 0.001). The intraindividual difference of simultaneous processing was significantly lower than that of planning (*p* = 0.002).

**Table 4:** Post-hoc multiple comparison of the CAS scores using Tukey’s test (n = 30).

Difference of levels		Difference of means	95%CI Lower, upper	*P* value
**Standard score**				
Planning	Successive	13.5	4.0, 23.0	0.002
Attention	Successive	9.6	0.1, 19.0	0.047
Simultaneous	Successive	4.6	− 4.9, 14.0	0.593
Planning	Simultaneous	8.9	− 0.5, 18.4	0.073
Planning	Attention	3.9	− 5.5, 13.4	0.702
Attention	Simultaneous	5.0	− 4.5, 14.5	0.518
**Intraindividual difference**				
Planning	Successive	13.5	7.3, 19.7	< 0.001
Attention	Successive	9.6	3.4, 15.8	< 0.001
Simultaneous	Successive	4.6	− 1.6, 10.8	0.224
Planning	Simultaneous	8.9	2.7, 15.1	0.002
Planning	Attention	3.9	− 2.3, 10.1	0.351
Attention	Simultaneous	5.0	− 1.2, 11.2	0.157

*CAS* Das Naglieri cognitive assessment system, *CI* confidence interval.

The mean value of the intraindividual difference in successive processing was lower in those with SPECT stage 2 than in those with non-stage 2 (− 7.6 ± 8.6 vs − 6.3 ± 8.9), although the difference was not significant.

The proportions of patients who exhibited significant intraindividual differences in each measure of the CAS and WISC-IV were as follows: planning (0/30 or 0%), attention (3/30 or 10%), simultaneous processing (4/30 or 13.3%), and successive processing (11/30 or 36.7%) for the CAS; and VCI (3/30 or 10%), PRI (5/30 or 16.7%), PSI (4/30 or 13.3%), and WMI (6/30 or 20%) for the WISC-IV.

## Discussion

Our study had three main findings. First, children with MMD exhibited selective within-individual weaknesses in successive processing as measured by the CAS. Second, intelligence measured with the WISC-IV was within the normal range with or without infarction. Third, no significant variability across the intellectual domains of the WISC-IV was observed, although the score for the working memory index was the lowest. The first finding is novel because CAS has not been applied to children with MMD, except for in a pioneering case report written in Japanese^26^. Furthermore, we used both the CAS and WISC to assess children with MMD and showed that the CAS detected selective neurocognitive weaknesses that were not detected by the WISC-IV.

Successive processing in the CAS is thought to be similar in concept to the WMI in the WISC-IV as both involve the auditory recall of a sequence. The successive processing scale of the CAS comprises three tasks: immediate recall of a series of words (e.g. cat-flower-book); repetition of sentences with no actual meaning (e.g. white is blue); and sentence questions as syntactic comprehension (e.g. the blue yellows the green, and who yellows the green?). The WMI in the WISC-IV comprises digit span and letter-number sequencing, whereas successive processing in the CAS involves language recall. Das et al., one of the groups that developed the CAS, has explained that successive processing involves the articulatory loop of working memory, as proposed by Baddeley^27^. Furthermore, a validation study on the Japanese version of the WISC-IV also revealed a high correlation between the successive processing scale of the CAS and the WMI of the WISC-IV^22^. Therefore, it is reasonable to suggest that successive processing reflects a form of verbal working memory function. Further studies are required to determine which components of working memory are associated with neurocognitive weaknesses in children with MMD.

Interestingly, our result of selective weakness in successive processing corresponds to those of a previous study. Hsu et al. reported that selective impairment in immediate word recall, as measured by the Word List Test in the Wechsler Memory Scale, might be a characteristic of MMD children with younger onset and prolonged symptom duration^17^. The successive processing scale of the CAS, which requires the working memory of language, might better reflect the cognitive skills required in daily life and the difficulties experienced by patients, such as inability to follow schoolwork, trying to learn too many things simultaneously, and poor attention. Our results also suggest that such difficulties could occur even if overall intelligence, as defined by the full scale scores, is high. Alternatively, the WMI of the WISC-IV, which comprises only digit span, may simply be impractical and unsuitable for detecting neurocognitive characteristics in children with MMD.

Kazumata et al. have suggested that verbal function is relatively preserved in MMD children^23^, which is inconsistent with our results. However, one reason for why the verbal working memory tasks of the CAS were more affected than the digit span task of the WISC in our study is that the former task is more complex and difficult because it requires not only basic working memory ability but also semantic processing ability. Therefore, our results are not inconsistent with the suggestion of Kazumata et al. In addition, logically, whether verbal function or non-verbal function is more impaired is defined by the laterality of disease involvement and may be less related to working memory function per se. Although the relatively complex working memory tasks of the CAS seem suitable for MMD children, the optimal working memory task for detecting working memory weaknesses specific to children with MMD remain to be ascertained. This issue should be addressed in future studies.

Selective impairment of neurocognitive domains has been attributed to hypoperfusion in specific vascular territories that are predominantly affected in MMD. Arterial stenosis in MMD involves the internal carotid artery by definition and thus causes ischemia of the frontal lobe^28^. Numerous studies have demonstrated a close relationship between the dorsolateral prefrontal cortex and executive functions, including working memory^29,30^. Moreover, Lezak et al. described that the prefrontal lobe is involved in short-term memory^31^. Therefore, our finding of verbal working memory disturbance in MMD is consistent with brain dysfunctions typically reported in MMD patients. However, further studies on cerebral perfusion imaging are required to clarify the relationship between working memory and previously reported cerebral blood flow changes in children with MMD^10,23^.

The fact that the scores of the WISC-IV fell within the normal range in our patients is consistent with previous studies that showed average to above average overall intelligence in children with MMD^17,32–35^ These results may be interpreted as an underestimation of within-individual variability in assessments using the WISC, given that variability is not measured by this scale. To address this question, we applied the method used for calculating intraindividual differences in the CAS to the WISC-IV and found no significant variability among the four measures, although the WMI score was the lowest. These findings suggest that the WISC-IV is less able to detect characteristics of neurocognitive deficits in children with MMD.

The present study has several limitations. First, our study population was relatively small. Although our study is one of the given the rarity of the disease, larger studies are needed. Nevertheless, our study design of using two standardized tests that are rarely applied to children demonstrates some validity of our results on neurocognitive profiles. Second, our study had the potential to have been contaminated by selection bias because we only included preoperative patients. The exclusion of children under 5 years of age, for whom neuropsychological testing is inapplicable, or those who required acute surgery because of unstable symptoms may overestimate our results and limit their generalizability. Finally, this study did not include a control group.

## Conclusions

Our assessment of the CAS suggests that a selective within-individual weakness in successive processing, a type of working memory, may be a characteristic of children with MMD. The CAS was more sensitive in detecting this selective neurocognitive weakness than the WISC-IV in children with MMD. Further studies are required to determine whether the CAS has value for clinical use in supporting the education and daily life of children with MMD.
